# Publication Trends of Pediatric and Adult Randomized Controlled Trials in General Medical Journals, 2005–2018: A Citation Analysis

**DOI:** 10.3390/children7120293

**Published:** 2020-12-15

**Authors:** Michael L. Groff, Martin Offringa, Abby Emdin, Quenby Mahood, Patricia C. Parkin, Eyal Cohen

**Affiliations:** 1 Department of Epidemiology and Biostatistics, Schulich School of Medicine and Dentistry, University of Western Ontario, London, ON N6A 5C1, Canada; mgroff@uwo.ca; 2 Child Health Evaluative Sciences, Hospital for Sick Children Research Institute, Toronto, ON M5G 0A4, Canada; martin.offringa@sickkids.ca (M.O.); abby.emdin@sickkids.ca (A.E.); patricia.parkin@sickkids.ca (P.C.P.); 3 Department of Pediatrics, University of Toronto, Toronto, ON M5S 1A1, Canada; 4 Institute of Health Policy, Management, and Evaluation, University of Toronto, Toronto, ON M5T 3M6, Canada; 5 Division of Neonatology, The Hospital for Sick Children, Toronto, ON M5G 1X8, Canada; 6 Global Child Health, Hospital for Sick Children Research Institute, Toronto, ON M5G 0A4, Canada; 7 Division of Hospital Library and Archives, The Hospital for Sick Children, Toronto, ON M5G 1X8, Canada; quenby.mahood@sickkids.ca; 8 Division of Pediatric Medicine, The Hospital for Sick Children, Toronto, ON M5G 1X8, Canada; 9 Edwin S.H. Leong Centre for Healthy Children, University of Toronto, Toronto, ON M5G 0A4, Canada

**Keywords:** pediatrics, child, randomized controlled trials, clinical trials, publication characteristics, bibliometrics, publishing, publications, journal impact factor, social media

## Abstract

Policy has been developed to promote the conduct of high-quality pediatric randomized controlled trials (RCTs). Whether these strategies have influenced publication trends in high-impact journals is unknown. We aim to evaluate characteristics, citation patterns, and publication trends of pediatric RCTs published in general medical journals (GMJs) compared with adult RCTs over a 13-year period. Studies were identified using Medline, and impact metrics were collected from Web of Science and Scopus. All RCTs published from 2005–2018 in 7 GMJs with the highest impact factors were identified for analysis. A random sample of matched pediatric and adult RCTs were assessed for publication characteristics, academic and non-academic citation. Citations were counted from publication until June 2019. Among 4146 RCTs, 2794 (67.3%) enrolled adults, 591 (14.2%) enrolled children, and 761 RCTs (18.3%) enrolled adult and pediatric patients. Adult RCTs published in GMJs grew by 5.1 publications per year (95% CI: 3.3–6.9), while the number of pediatric RCTs did not show significant change (−0.4 RCTs/year, 95% CI: −1.4–0.6). Adult RCTs were cited more than pediatric RCTs (median(IQR): 29.9 (68.5–462.8) citations/year vs. 13.2 (6.8–24.9) citations/year; *p* < 0.001); however, social media attention was similar (median(IQR) Altmetric Attention Score: 37 (13.75–133.8) vs. 26 (6.2–107.5); *p* = 0.25). Despite policies which may facilitate conduct of pediatric RCTs, the publishing gap in high-impact GMJs is widening.

## 1. Introduction

Randomized controlled trials (RCTs) are regarded as the “gold standard” in interventional research design [1]. Results of RCTs, when used judiciously in an evidence-based context, have potential to improve health outcomes [2,3]. Childhood illness comprises a significant portion of global disease burden, with persons aged 0–19 years old comprising approximately 26.2% of all-cause disability-adjusted life years (DALYs) as of 2019 [4]. Despite this, there exists a mismatch between pediatric RCT activity and pediatric disease burden [5]. Previous studies from over a decade ago have noted this paucity of pediatric RCTs in published literature, particularly in general medical journals [6,7,8].

General medical journals (GMJs) are important sources of information for family physicians, generalists and subspecialists. These journals publish high-quality research which is widely read, cited, and disseminated; as of 2019, the top five GMJs all have journal impact factors higher than any pediatric journal [9]. In high-impact GMJs, recent studies have documented a paucity in the publication of RCTs conducted in low- and middle-income countries as well as RCTs addressing diseases of poverty [10,11], diseases which disproportionately affect children [12]. This suggests that high-impact GMJs may underreport trials relating to diseases of childhood.

We previously reported that the publication of pediatric RCTs in GMJs did not increase from 1985 to 2004, whereas the publication of adult RCTs increased substantially over the same period [8]. Since that time, a number of legislative initiatives in the US and Europe to promote the conduct of pediatric clinical trials have been implemented [13,14,15]. While there is considerable evidence that these policies have been effective in increasing the availability of evidence-based therapeutics for children [16], it is unknown whether these policies have led to an increased initiation, completion, or publication of high-quality pediatric RCTs in GMJs [17]. The aim of this study was to compare the numbers of pediatric and adult RCTs published in GMJs in the period 2005 to 2018, and to compare these trials’ characteristics and impact.

## 2. Materials and Methods

We searched GMJs indexed under the “Medicine—General and Internal” category in Journal Citation Reports [9]. Excluded were those GMJs focused solely on adult internal medicine (e.g., Annals of Internal Medicine) and reviews (e.g., Cochrane Database of Systematic Reviews). We selected the seven highest ranked GMJs ranked by two-year 2017 journal impact factors: New England Journal of Medicine (79.26); Lancet (53.25); Journal of the American Medical Association (47.66); British Medical Journal (23.56); PLoS Medicine (11.68); BMC Medicine (9.09); Canadian Medical Association Journal (6.82); we chose this cut-off to ensure relatively new high-impact open-access journals (PLoS Medicine and BMC Medicine) were included as well.

We defined pediatric and adult RCTs as follows: pediatric RCTs were those RCTs in which the population of interest, and corresponding intervention and comparison groups, were all patients less than 18 years of age. Adult RCTs were defined as RCTs including exclusively adult patients (≥18 years old) in their population of interest as well as their intervention and comparison groups. Mixed RCTs were defined as RCTs with intervention and/or comparison groups including pediatric (<18 years old) and adult (≥18 years old) patients.

RCTs in the included journals were identified using an electronic search strategy and refined using a machine learning algorithm, RobotSearch [18]. An Ovid Medline search strategy (1 January 2005–1 June 2018) was developed by the study team’s information science specialist (QM). The Cochrane Highly Sensitive Search, optimized for sensitivity and specificity [19], was used to ascertain RCTs at the search strategy level. This search strategy also stratified RCTs by journal, index year of publication, and age cohort (pediatric, adult, mixed). After comparing Medline age search queries [20,21], we deemed built-in age filters were sensitive enough for our purposes with a sensitivity of 94% compared with a hand search [20]. The electronic search strategy is presented in full in Table 1.

Non-randomized study designs can be erroneously classified as RCTs in Ovid Medline [22,23]. To improve the specificity of our search, we used a validated machine learning algorithm (RobotSearch) to further refine our ability to accurately identify RCTs. RobotSearch uses a machine learning algorithm to yield a highly sensitive (98.5%) and specific (87.6%) method to screen the results of our search strategy and effectively identify RCTs in our search results [18]. Accuracy of RobotSearch was verified by a hand-search of all 97 publications detected by our search strategy from JAMA in the journal year 2018. RobotSearch had a sensitivity of 97.0% and a specificity of 83.8% when compared to a hand search.

We measured pediatric and adult RCT quantity as the number of indexed RCTs in each calendar year across all seven selected journals. In addition, we assessed the number of RCTs over the study period for each individual journal.

We also assessed publication characteristics and impact for a subset of pediatric and adult RCTs. A sample of 10% of included pediatric RCTs ascertained in each electronic search was selected using a random number generator and matched (1:1) to an adult RCT published in the same journal and year.

The following RCT characteristics were collected: publishing journal, RCT design (parallel, cluster, crossover, factorial), hypothesis type (superiority, noninferiority, equivalence), “positive” or “negative” results as reported by the authors, sample size, number of centres in multicentre trials, number of countries involved in multinational trials, continents involved in the RCT (Africa, Asia, Europe, North America, Oceania or South America), degree of blinding of intervention (no blinding, single-blind, double-blind or more), and industry funding (yes/no). RCTs were classified as being conducted on a continent if at least one study centre was located on that continent.

In cases where centre location and other characteristics were ambiguous in the trial manuscript and included supplements, clinical trial registries provided information that obviated the need for further investigation. Clinical trial registries used for additional clarification included ClinicalTrials.gov, International Standard Randomised Controlled Trials Number (ISRCTN) registry, Karolinska Clinical Trial Registration, Australian New Zealand Clinical Trials Registry (ANZCTR), and the Netherlands Trial Register (NTR). While many trials were registered in other national trial databases, these trials were all either registered in one of the above registries as well, or provided information was unambiguous enough to not warrant further investigation.

Study sample size was collected because it may serve as a proxy for a study’s susceptibility to random error. Small sample sizes may result in the overestimation or underestimation of treatment effects [24,25]. We collected data on how many centres participated in each study and where they were located; multicentre and particularly multinational trials are indicators, at least in part, of the generalizability of a trial. We defined RCTs with double-blinding or greater as ones blinding the patient and any other parties, including the investigators, outcomes assessor, data collectors, and data analysts, among other possible parties.

We defined “industry-funded” RCTs as those with financial compensation reported from a for-profit organization, as opposed to institutional grants and governmental organizations. We explored the relationship between funding source and the authors conclusions in our sample, as industry funding has been previously shown to be associated with publication and interpretation biases of results [26,27,28]. If a relationship exists in either adult or pediatric RCTs this may imply a form of publication bias or biased interpretation of trial results [29].

RCT impact was assessed using a variety of measures of academic and non-academic citation. These measures were collected through Web of Science and Scopus. The Altmetric Attention Score and PlumX Index account for references to the study from various forms of media. The PlumX measure is a count of all types of references, stratified across five different domains. The Altmetric Attention Score provides a measure of the amount of attention an article has received since publication [30]. This measure is presented as a weighted count of all types of citation/media categories, each receiving a different weighting. Web of Science was used to provide a conservative citation count. Web of Science, generally speaking, has a narrower coverage of biomedical journals than Scopus and will therefore give a more conservative citation count [31,32]. As there will be variation in citation estimates based on the database used, we have opted to analyze both Web of Science and Scopus data. Altmetric data from Web of Science and PlumX data from Scopus were used to provide estimates of non-academic citation. Scopus indexes a broader range of journals than Web of Science, although Web of Science and Scopus have considerable overlap [31,32]. We collected data on citation numbers and frequencies from Web of Science and Scopus. Analyses were conducted using Web of Science and Scopus data; however, results did not differ between the two. We elected to use Web of Science data for the primary analysis.

The numbers of pediatric and adult RCTs per year published across the seven selected GMJs, 2005 to 2018, were assessed using linear regression. An interaction term was used to determine whether the (change in) publication rate of pediatric RCTs versus adult RCTs was different. Given that some trials report on combined pediatric and adult populations, a sensitivity analysis was conducted with the assumption that all trials indexed as including both pediatric and adult patients were either pediatric or adult trials, respectively.

Pediatric and adult RCT characteristics were compared using chi-squared tests for nominal and ordinal outcomes. Mann-Whitney tests were conducted to assess for differences in sample size and to compare impact scores of pediatric and adult RCTs. A sensitivity analysis using Scopus data in place of Web of Science data for citation number, average citation frequency, and citation data was conducted and no notable differences were found. All *p*-values were 2-tailed, and values <0.05 were considered significant. Analyses were conducted using STATA 15.0 (StataCorp, College Station, TX, USA).

## 3. Results

### 3.1. Search Results

**Figure 1:**
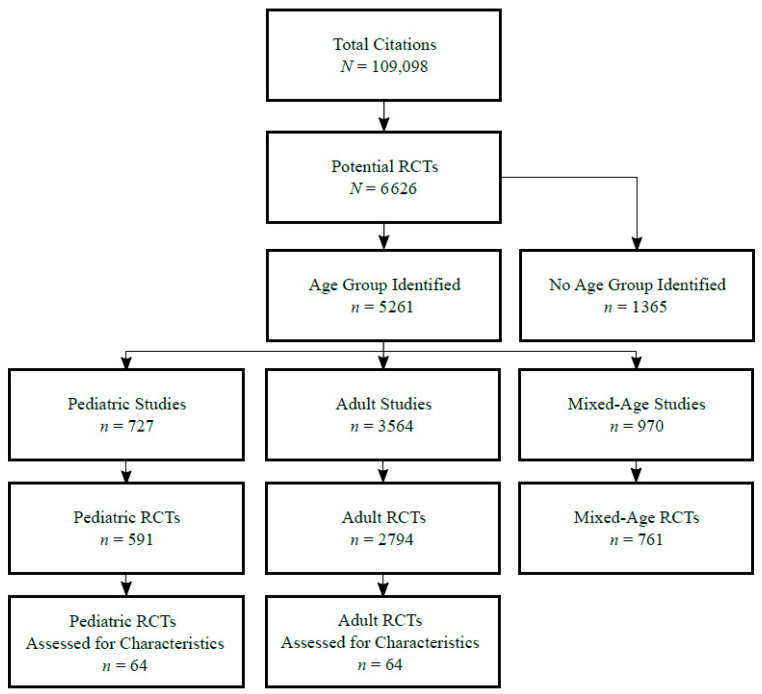
Study selection flow diagram stratified by age group of study. RCTs: randomized controlled trials.

Figure 1 summarizes search results at each stage by age group(s) included in the study. Among 109,098 citations in the seven GMJs from 2005 to 2018, 6626 (6.1%) were classified as RCTs by our electronic search strategy. The total number of RCTs was reduced to 4146 (3.8%) with RobotSearch, constituting the sample for further analysis. Among these, 2794 RCTs (67.3%) recruited adult patients only, 591 RCTs (14.2%) with solely pediatric patients, and 761 RCTs (18.3%) including both adult and pediatric patients. Among the seven GMJs, the Journal of the American Medical Association (JAMA) published the most adult RCTs (*n* = 717), the Lancet published both the most pediatric RCTs (*n* = 275) and the most mixed (both adult and pediatric) RCTs (*n* = 275). In total, 64/591 (10.8%) pediatric RCTs and 64/2794 (2.3%) adult RCTs were randomly selected for further assessment.

**Figure 2:**
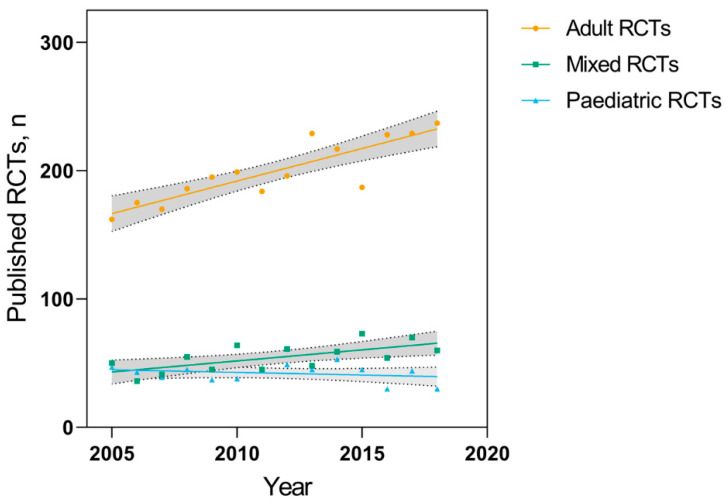
Number of adult, pediatric, and mixed-age RCTs published per year from 2005–2018 in seven high-impact general medical journals (GMJs). Shaded areas represent 95% confidence bands for each linear regression.

The number of adult, pediatric, and mixed RCTs per year from 2005 to 2018 is shown overall (Figure 2) and by each individual journal (Table 2). The overall number of adult RCTs published each year increased by a mean of 5.1 RCTs per year (95% CI: 3.3 to 6.9, *p* < 0.001), while the number of pediatric RCTs did not change substantially (−0.4 RCTs per year, 95% CI: −1.4 to 0.6, *p* = 0.44). RCTs including both pediatric and adult patients also increased by a mean of 1.7 RCTs per year (95% CI: 0.5 to 2.9, *p* = 0.01).

These numbers did not change substantially in our sensitivity analyses. Classifying all RCTs including both pediatric and adult patients as adult RCTs, increased the average change in the number of RCTs published in this group to 6.8 RCTs per year (95% CI: 5.1 to 8.5; *p* < 0.001). Classifying all RCTs including both pediatric and adult patients as pediatric RCTs resulted in no significant change in the mean number of RCTs published per year (1.3 RCTs per year, 95% CI: −0.5 to 3.1; *p* = 0.14).

### 3.2. RCT Characteristics and Impact

#### 3.2.1. Characteristics

Table 3 compares characteristics of adult (*n* = 64) and pediatric (*n* = 64) RCT samples. Adult and pediatric RCTs had similar proportions of parallel-group RCTs, superiority hypotheses, and positive results.

Pediatric and adult RCTs had similar median sample sizes (467 (interquartile range, (IQR: 145 to 3021) vs. 495 (IQR: 243 to 2024), *p* = 0.49). Pediatric RCTs were about half as likely to be multinational compared with adult RCTs (32.6% vs. 67.4%, *p* = 0.002). Single blinding was more common in pediatric RCTs compared with adult RCTs (29.7% vs. 9.4%, *p* = 0.004). The proportions of pediatric and adult multicentre and industry funded RCTs were similar.

Pediatric RCTs were more likely to be conducted in Africa (28.1% vs. 9.4%, *p* = 0.007) and Oceania (23.4% vs. 7.8%, *p* = 0.02), while adult RCTs were more likely to be conducted in Europe (70.3% vs. 34.4%, *p* < 0.001), and North America (59.4% vs. 25.0%, *p* < 0.001). All pediatric RCTs from Africa were conducted exclusively in Africa. All but four of these RCTs address an infectious disease such as malaria (9/18), rotavirus (3/18), HIV (1/18), and streptococcus pneumoniae (1/18). The other four focused on malnutrition (2/18), anemia (1/18), and all-cause pneumonia (1/18). All adult RCTs with centres in Africa also had centres in Europe, North America, and South America. These trials (*n* = 6) each covered a different non-infectious condition.

#### 3.2.2. Impact

Table 4 summarizes measures of academic and non-academic citation. Using Web of Science, adult RCTs were cited more often compared to pediatric RCTs (median (IQR): 29.9 citations/year (IQR: 12.5 to 53.3) vs. 13.2 citations/year (IQR: 6.9 to 24.9), *p* < 0.001). Changing databases did not change these results; adult RCTs were also cited more often compared to pediatric RCTs using Scopus (median (IQR): 33.7 citations/year (13.9 to 57.1) vs. 14.2 citations/year (13.9 to 57.1), *p* < 0.001). Adult trials also had higher PlumX “Citations” scores. Altmetric scores for adult RCTs were similar to pediatric RCTs (median (IQR): 37.0 (13.8 to 133.8) vs. 26.0 (6.2 to 107.5), *p* = 0.25).

## 4. Discussion

Our study found that adult RCTs published in high-impact GMJs are continuing to increase in number with no change in the number of published pediatric RCTs. RCT characteristics were similar between adult and pediatric trials, although adult RCTs were more likely to be multinational. Adult RCTs were more frequently cited in academic publications, but not in other media. Despite important policies to promote the conduct of RCTs in children implemented in the past two decades, our results suggest there may be ongoing barriers to the publication of high-quality pediatric RCTs in GMJs.

Our findings are similar to a report of the five highest impact GMJs conducted by our study team from 1985–2004 [8]. We found similar growth in the number of RCTs involving adults (5.1 RCTs per year (95% CI: 3.3 to 6.9) in the current study vs. 4.7 RCTs per year (95% CI 3.6 to 5.8)), and similar flatlining in the number of RCTs involving children (−0.4 RCTs per year (95% CI: −1.4 to 0.6) vs. 0.4 RCTs per year (95% CI −0.0 to 0.9)). Taken together, the results of these two studies suggest that the gap between the annual number of published adult and pediatric RCTs in GMJs has continued to widen for over 30 years.

There are a number of possible explanations for our findings. First, legislative and other policy initiatives may have been ineffective at promoting the initiation, completion, or publication of pediatric trials. Initiatives have aimed to promote the conduct of pediatric RCTs by giving industry sponsors increased patent durations and vouchers for expedited product review. For some interventions, regulations set out by these policies mandate conduct of a pediatric RCT alongside an adult RCT unless a waiver is warranted. Examples of these policies include the Best Pharmaceuticals for Children Act and Pediatric Research Equity Act—both now permanently under the Food and Drug Administration Safety and Innovation Act of 2012 in the United States [14,15,33] and the 2007 Pediatric Regulation in Europe [13], respectively. Although there have been a greater number of interventions evaluated for safety and efficacy in children [16,33], industry and governmental stakeholders may still lack adequate incentives to sponsor trials to completion. For instance, the Pediatric Rare Diseases Voucher Program, which provides direct incentives for the conduct of pediatric RCTs aiming to treat rare diseases, has not been associated with any increase in the rate of pediatric drug testing [17] and has since undergone revisions [34].

When a pediatric RCT is initiated, unique aspects of pediatric RCTs such as nuances of informed consent may lead to poor recruitment. An inability to meet recruitment targets in pediatric trials has been associated with the non-submission of RCTs for publication [35]. However, evidence suggests poor recruitment is not independently attributable to the pediatric setting [36]. There may also be ethical and logistical barriers to conducting high-quality pediatric RCTs such as unique aspects of consent/assent in pediatric trials [37] in addition to the common struggle of poor recruitment [35,36]. Consent rates in pediatric RCTs have improved over time, but are still considered suboptimal [38,39]. However, it is noteworthy that our findings are qualitatively similar to findings before the institution of policy changes to minimize these barriers [8], suggesting other factors may be at play. Publication may be more challenging because pediatric RCTs may have suboptimal quality [7,40,41], although comparative studies consistently found no detectable difference in quality between pediatric and adult RCTs [6,7,8]. While we did not use a standardized quality assessment tool, we did find differences in some indicators of study quality or rigor (e.g., blinding) but not others (e.g., sample size, multicentre trials).

There could also be barriers at the level of GMJs. Authors of pediatric RCTs may not submit trials to GMJs and/or the editors of GMJs may be less likely to accept them. Authors of pediatric RCTs may not submit their RCTs for publication due to factors such as perceptions about statistical or clinical significance, agreement with their initial hypotheses, and authors’ perceptions of the trials’ likelihood of acceptance by high-ranking GMJs [42]. Because of these perceptions, authors of pediatric studies may prefer submitting their works to smaller, more “pediatric-friendly” journals. However, our findings are also similar to results from a report of a large difference between the growth of adult and pediatric RCTs per year (90.5 RCTs/year vs. 16.9 RCTs/year) from 1985–2005 in general pediatric and specialty journals, suggesting that our findings may not be unique to GMJs [6].

The editors of GMJs may prefer publishing research of general medical interest, which may be more focused on adult-onset disease, particularly those prevalent in high-income countries with an aging population. Conversely, pediatric issues such as infant mortality, infectious diseases, and diseases of poverty may have larger impacts in lower income regions of the world [12]. Pediatric RCTs conducted in low- and middle-income countries also face several unique challenges due to the importance of community engagement, power differentials, medical mistrust, and other ethical issues. These challenges may act as barriers to the production of high-quality pediatric RCTs from low- and middle-income countries [43]. We also observed that pediatric RCTs conducted in Africa frequently study infectious diseases and diseases of poverty such as malnutrition. This may explain our finding of more pediatric RCTs conducted in global regions with a larger concentration of low-income countries (e.g., Africa), and published in GMJs with a more deliberate focus on global health (e.g., the Lancet). The promotion of RCTs in a global health setting may be one strategy to increase publication of pediatric RCTs, although such studies may not address important health issues affecting children in high-income and middle-income countries. Another explanation is that journals may preferentially publish adult RCTs due to higher citation counts and a perceived higher impact. Adult RCTs were more frequently cited in GMJs, which may reflect study quality or impact. However, a relationship between citations and study quality has not been consistently reported [44], and academic citation metrics have been criticized for their poor ability to measure true research impact [45]. Previous studies have not compared adult and pediatric trials in terms of broader impact, including social media. Our finding of similar social media scores in Altmetric suggests that the non-academic attention received by pediatric RCTs published in GMJs may not differ substantially from adult RCTs.

One of the unique strengths of our study is our use of a study selection strategy which combined a highly sensitive search strategy for detecting RCTs [19] with a machine learning algorithm, RobotSearch [18]. Although this RCT filtering technology has not been used much previously, comparison with manually abstracted data from one journal year found that this method had high, albeit imperfect, precision. While age indexing was suboptimal, sensitivity analyses showed that even if all mixed RCTs are misclassified this would not have affected our findings.

This study has a few important limitations. First, we limited our analysis to GMJs. The majority of pediatric RCTs—including many high-quality trials—are published in general pediatric or pediatric subspecialty journals. Second, our study was limited to seven GMJs with the highest impact factor, meaning RCTs published in these journals are likely not representative of all GMJs, and certainly not all pediatric and adult RCTs. Third, we used a well-accepted cut-off of 18 years to distinguish between pediatric and adult RCTs [41]. Although this age cut-off is widely used to define pediatric populations, it is to some extent dependent on the geographical region of study. If we used a different age cut-off, we may have found somewhat different findings. Fourth, while the addition of the machine learning algorithm to our search strategy did increase efficiency so that we could identify a large number of studies, it did not have perfect sensitivity. Although we may have missed some trials, our full-text review process ensured optimal specificity. Fifth, our secondary analyses of study characteristics and impact were underpowered to detect small differences between groups due to the relatively small numbers of RCTs compared. Lastly, we assessed RCT impact using traditional academic as well as more contemporary non-academic metrics, but such metrics are approximate estimates of true impact. The influence of the RCTs on clinical practice and policy could not be assessed.

## 5. Conclusions

Despite policies which may promote the conduct of high-quality pediatric RCTs, we found no substantial increase in pediatric RCTs published in high-impact GMJs, while the number of published RCTs in adults has continued to grow, a gap that has now persisted for over 30 years.

## Figures and Tables

**Table 1:** Ovid Medline search strategy for locating indexed pediatric, adult, and mixed randomized controlled trials (RCTs).

Search Strategy	Description
1. randomized controlled trial.pt.	Cochrane Highly Sensitive Search (Steps 1–10).
2. controlled clinical trial.pt.	
3. randomized.ab.	
4. placebo.ab.	
5. clinical trials as topic.sh.	
6. randomly.ab.	
7. trial.ti.	
8. 1 or 2 or 3 or 4 or 5 or 6 or 7	
9. exp animals/not humans.sh.	
10. 8 not 9	
11. (Jama or “Journal of the American Medical Association”).jn.	Limit by journal. *
12. limit 11 to yr = ”2018”	Limit by year. *
13. limit 12 to (address or comment or editorial or letter or observational study or meta-analysis or review or “systematic review”)	Filter out indexed addresses, comments, editorials, letters, observational studies, meta-analyses, reviews, and systematic reviews (Steps 13–14).
14. 12 not 13	
15. 10 and 14	All studies meeting above criteria (base RCT number).
16. limit 15 to “all child (0 to 18 years)”	Limit to studies indexed as including children.
17. limit 15 to “all adult (19 plus years)”	Limit to studies indexed as including adults.
18. 15 not (16 or 17)	Studies not indexed by any age group.
19. 16 not 17	Studies indexed as only including pediatric patients.
20. 17 not 16	Studies indexed as only including adult patients.
21. (16 or 17) not (19 or 20)	Studies indexed as including both pediatric and adult patients.

* Example shows the search query for JAMA in the year 2018. This search strategy was repeated for all included journals using their abbreviation (where applicable) and their full name as well as each calendar year from 2005 to 2018, inclusive.

**Table 2:** Number of published RCTs by age group (pediatric, adult), publishing journal, and year of publication.

		Year of Publication													
Journal	Change in RCTs/year, β (95% CI)	2005	2006	2007	2008	2009	2010	2011	2012	2013	2014	2015	2016	2017	2018
**Adult RCTs**															
**Lancet**	2.4 (0.7; 4.0)	45	33	49	56	63	62	59	45	54	62	40	80	73	85
**NEJM**	0.9 (0.2; 1.6)	38	46	45	50	45	55	49	56	55	52	56	44	49	61
**JAMA**	1.9 (0.5; 3.2)	45	50	40	46	44	36	21	50	63	70	56	59	71	56
**BMJ**	–1.4 (–2.3; –0.5)	25	32	24	25	34	32	27	90	30	13	15	17	9	15
**PLoS Medicine**	0.8 (0.3; 1.2)	1	3	4	5	2	2	5	3	3	6	4	16	12	11
**BMC Medicine**	0.9 (0.4; 1.3)	2	3	3	2	1	5	7	7	15	8	15	7	13	9
**CMAJ**	–0.3 (–0.7; 0.1)	6	8	5	2	6	5	1	8	9	6	1	5	2	0
**TOTAL**	5.1 (3.3; 6.9)	162	175	170	186	195	197	169	259	229	217	187	228	229	237
**Pediatric RCTs**															
**Lancet**	–0.6 (–1.1; 0.0)	23	15	11	12	11	8	11	14	15	8	17	10	8	7
**NEJM**	0.2 (–0.2; 0.6)	2	10	7	11	3	10	10	12	9	9	8	9	8	9
**JAMA**	0.2 (–0.2; 0.5)	5	6	5	7	6	7	4	8	9	13	7	3	10	6
**BMJ**	–0.8 (–1.2; –0.4)	11	8	15	13	9	8	12	10	7	8	7	0	1	2
**PLoS Medicine**	0.3 (–0.0; 0.6)	3	1	1	1	3	3	8	4	1	9	3	5	7	3
**BMC Medicine**	0.2 (0.0; 0.4)	0	2	2	2	3	0	1	1	2	4	1	3	6	3
**CMAJ**	0.0 (–0.2; 0.2)	3	1	0	1	2	2	0	0	2	2	2	0	4	0
**TOTAL**	–0.4 (–1.4; 0.6)	47	43	41	47	37	38	46	49	45	53	45	30	44	30

NEJM: New England Journal of Medicine; JAMA: Journal of the American Medical Association; BMJ: British Medical Journal; CMAJ: Canadian Medical Association Journal.

**Table 3:** Characteristics of a stratified random sample of pediatric and adult RCTs in seven general medical journals, 2005–2018.

	Pediatric RCTs *n* = 64	Adult RCTs *n* = 64	*p*-Value
**Journal, *n* (%)**			
**Lancet**	21 (33)	18 (28)	0.23
**NEJM**	17 (27)	26 (41)	
**JAMA**	8 (12)	10 (16)	
**BMJ**	11 (17)	8 (12)	
**PLoS Medicine**	3 (5)	0 (0)	
**BMC Medicine**	2 (3)	1 (2)	
**CMAJ**	2 (3)	1 (2)	
**Sample Size, median (IQR)**	467 (2876)	495 (1781)	0.49
**Trial design, *n* (%)**			
**Parallel**	54 (84)	55 (88)	0.72
**Cluster**	9 (14)	5 (8)	
**Factorial**	1 (2)	1 (2)	
**Crossover**	0 (0)	3 (3)	
**Hypothesis, *n* (%)**			
**Superiority**	55 (86)	60 (94)	0.21
**Non-inferiority**	7 (11)	4 (6)	
**Equivalence**	2 (3)	0 (0)	
**Blinding, *n* (%)**			
**Double+**	30 (47)	37 (58)	0.01
**Single**	19 (30)	6 (9)	
**Open label**	15 (23)	21 (33)	
**Results**			
**Positive**	44 (69)	39 (61)	0.36
**Negative**	20 (31)	25 (39)	
**Multicentre trials, *n* (%)**			
**Yes**	51 (80)	56 (88)	0.23
**No**	13 (20)	8 (12)	
**Multinational trials, *n* (%)**			
**Yes**	16 (25)	33 (52)	0.002
**No**	48 (75)	31 (48)	
**Continent, *n* (%)**			
**Europe**	22 (34)	45 (70)	0.002
**Africa**	18 (28)	6 (9)	
**North America**	16 (25)	38 (59)	
**Asia**	9 (14)	15 (23)	
**Oceania**	5 (8)	15 (23)	
**South America**	5 (8)	14 (22)	
**Industry funding, *n* (%)**			
**No**	36 (56)	36 (56)	>0.99
**Yes**	28 (44)	28 (44)	

**Table 4:** Measures of academic and non-academic impact in pediatric RCTs and adult RCTs.

	Pediatric RCTs	Adult RCTs	*p*-Value
**Citations (Web of Science)**	88.5 (42.5, 188.8)	157.5 (68.5, 462.8)	0.004
**Citations (Scopus)**	101.5 (51.8, 197.0)	168.5 (73.5, 562.8)	0.003
**Citation Frequency per year (Web of Science)**	13.2 (6.9, 24.9)	29.9 (12.5, 53.3)	<0.001
**Citation Frequency, per year (Scopus)**	14.2 (7.6, 27.0)	33.7 (13.9, 57.1)	<0.001
**Altmetric**	26.0 (6.2, 107.5)	37.0 (13.8, 133.8)	0.25
**PlumX-Citations**	102.0 (52.8, 199.2)	173.0 (73.2, 565.8)	0.005
**PlumX-Usage**	936.0 (534.0, 1535.5)	995.5 (433.2, 1676.2)	0.97
**PlumX-Capture**	191.5 (109.2, 364.0)	276.0 (112.0, 440.8)	0.18
**PlumX-Mentions**	1.0 (0.0, 2.0)	1.0 (0.0, 4.8)	0.15
**PlumX-Social**	16.0 (1.0, 92.0)	26.5 (1.2, 86.8)	0.49

All data are presented as median and interquartile range (IQR).
